# Characterization of 2,500 Patients with Heart Failure and Analysis of Their Optimal Medical Therapy: Insights from the AMERICCAASS Registry

**DOI:** 10.5334/gh.1418

**Published:** 2025-03-11

**Authors:** Alex David Sotomayor-Julio, Sebastián Seni-Molina, Juliana María Gutiérrez-Posso, Juan Andrés Muñoz-Ordoñez, Valeria Azcárate-Rodríguez, Hoover O. León-Giraldo, Eduardo R. Perna, Víctor Rossel, Daniel Quesada-Chaves, Mario Speranza, Mark H. Drazner, Walter Alarco, Alexander Romero-Guerra, Gabriel Frago, Daniela García Brasca, Álvaro Mauricio Quintero-Ossa, Javier Galeano Figueredo, Milton Lubeck Herrera, Antonella A. Ferrer, Ruddy Miguel García-Safadit, Freddy Pow-Chon-Long, Felix Nunura Arrese, Kwame van der Hilst, Silvia Carolina Lazo-Majano, Elisabeth Ashley Hardin, Orlando David Fernández-Flores, Gabriela Ormaechea-Gorricho, Luis Felipe Anhuaman-Atoche, Annia María Carrero-Vásquez, Andrés Ulate Retana, Pablo Hurtado Nuñez, Emilio Samael Peralta-López, Juan Esteban Gómez-Mesa, On Behalf of AMERICCAASS Research Group

**Affiliations:** 1 Servicio de Cardiología, Fundación Valle del Lili, Cali, Colombia; 2 Centro de Investigaciones Clínicas (CIC), Fundación Valle del Lili, Cali, Colombia; 3 Facultad de Ciencias de la Salud, Universidad Icesi, Cali, Colombia; 4 División de Insuficiencia Cardiaca e Hipertensión Pulmonar, Instituto De Cardiología J. F. Cabral, Corrientes, Argentina; 5 Servicio de Cardiología, Instituto Nacional del Tórax, Santiago, Chile; 6 Facultad de Medicina, Universidad de Chile, Santiago, Chile; 7 Servicio de Cardiología, Hospital San Vicente de Paul, Heredia, Costa Rica; 8 Departamento de Cardiología, Hospital Clínica Bíblica, San José, Costa Rica; 9 Division of Cardiology, University of Texas Southwestern Medical Center, Dallas, United States; 10 Servicio de Cardiología, Instituto Nacional Cardiovascular INCOR, Lima, Perú; 11 Servicio de Cardiología, Hospital Santo Tomas, Ciudad de Panamá, Panamá; 12 Unidad de Insuficiencia Cardiaca y Trasplante, Hospital Italiano de Córdoba, Córdoba, Argentina; 13 Departamento de Cardiología, Clínica Cardio VID, Medellín, Colombia; 14 Hospital de Clínicas –Facultad de Ciencias Médicas, Universidad Nacional de Asunción, Asunción, Paraguay; 15 Clínica HART, Quezaltenango, Guatemala; 16 Departamento de Cardiología, Unidad Cardiovascular Ferrer, Puerto Cabello, Venezuela; 17 Departamento de Cardiología, Centro Médico Siglo 21, San Francisco de Macorís, Republica Dominicana; 18 Departamento de Cardiología, Hospital Luis Vernaza, Guayaquil, Ecuador; 19 Heart Failure Clinic, Heart Institute of the Caribbean, Kingston, Jamaica; 20 Department of Cardiology, Thorax Center Paramaribo –Academic Hospital Paramaribo, Paramaribo, Suriname; 21 Departamento de Cardiología, Instituto Salvadoreño del Seguro Social, San Salvador, El Salvador; 22 Department of Internal Medicine, University of Texas Southwestern Medical Center, Dallas, United States; 23 Caja Petrolera de Salud, La Paz, Bolivia; 24 Departamento de Medicina Interna, Hospital de Clínicas ‘Dr. Manuel Quíntela’, Montevideo, Uruguay; 25 Departamento de Cardiología, Hospital María Auxiliadora, Lima, Perú; 26 Servicio de Cardiología, Hospital General Docente Enrique Cabrera, La Habana, Cuba; 27 Servicio de Cardiología, Hospital México CCSS, San José, Costa Rica; 28 Servicio de Cardiología, Hospital Carlos R. Huembes, Managua, Nicaragua; 29 Servicio de Cardiología, Instituto Nacional Cardiopulmonar, Tegucigalpa, Honduras; 30 Servicio de Cardiología, Cardio Medical Center, Tegucigalpa, Honduras

**Keywords:** Heart failure, Americas, optimal medical therapy, registries, characterization

## Abstract

**Introduction:**

Heart failure (HF) is a leading cause of hospitalization and mortality worldwide, emphasizing the critical role of optimal medical therapy (OMT) in improving patient outcomes. Despite extensive research, most scientific evidence regarding HF is gathered and studied in developed countries, leaving substantial knowledge gaps regarding HF in Latin America and the Caribbean.

**Objective:**

To characterize the sociodemographic and clinical profiles of HF patients and to assess their adherence to OMT in the Americas.

**Methods:**

The AMERICCAASS Registry is a prospective, observational, multicenter study, including patients aged 18 and older, both hospitalized and ambulatory, and diagnosed with HF. Sociodemographic and clinical data were collected from the first 2,500 patients to characterize the study population. Adherence to OMT was subsequently evaluated according to left ventricular ejection fraction (LVEF).

**Results:**

Among the 2,500 patients in the study, 36% were hospitalized and 64% were ambulatory. The median ages of the patients were 66.9 (hospitalized) and 66.3 years (ambulatory). Males made up 60.8% of hospitalized and 59.3% of ambulatory patients. The majority had HF with reduced LVEF (≤40%): 60.7% for hospitalized and 58.5% for ambulatory. The New York Heart Association (NYHA) functional class II predominated among ambulatory patients (67.9%), while NYHA functional class III predominated among hospitalized patients (46.6%). Only 21% of patients with reduced LVEF were receiving quadruple therapy, whereas 12.3% of patients with mildly reduced LVEF (41–49%) were on this treatment.

**Conclusion:**

The findings demonstrate that the sociodemographic and clinical profiles of HF patients in the Americas are broadly consistent with international reports. However, the low use of OMT observed in this population underscores gaps in adherence to current guidelines. These results highlight the need for targeted strategies to improve pharmacological treatment adherence to optimize health outcomes in this region.

## Graphical Abstract

**Figure:**
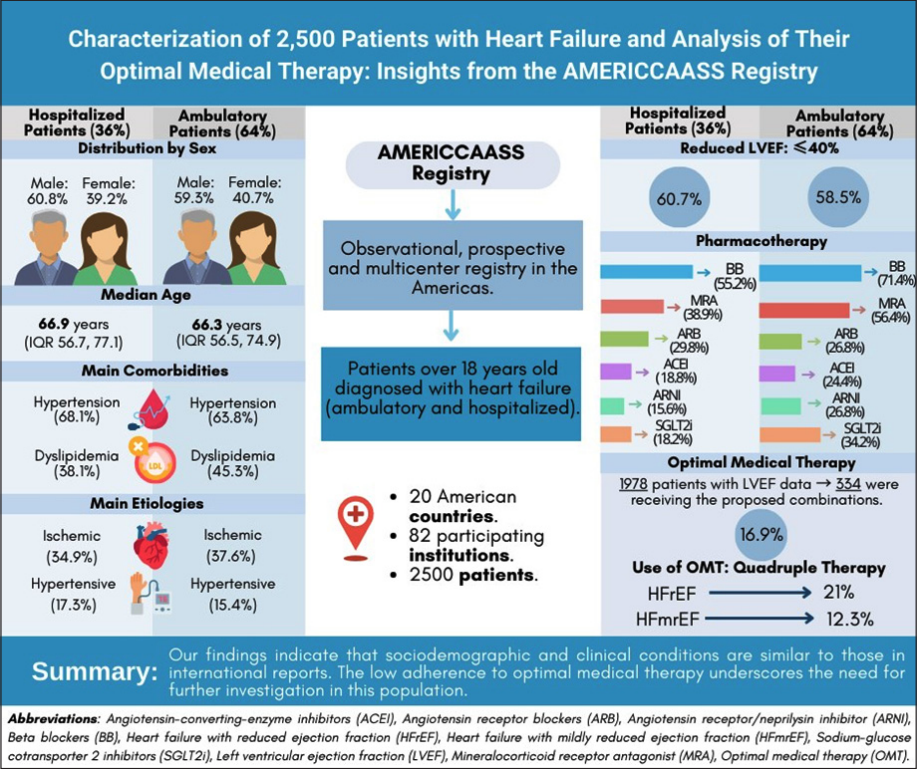


## Introduction

Heart failure (HF) is a significant public health problem, with a global prevalence estimated at 1–3% (1). In 2017, approximately 64.3 million people were affected, with the highest prevalence observed in Central Europe, North Africa, and the Middle East (1,2). Its prevalence is rising as a result of an aging population, and thanks to improvements in the management of acute cardiovascular events (1). This trend is reflected in hospitalization rates, where HF accounts for 1–2% of hospital admissions in Western countries (1). Its economic burden is considerable; in the US, HF-related expenditures reached $30.7 billion in 2012, with projections estimating an increase of up to 127% by 2030 (1,2,3,4).

Although 80% of the global burden of cardiovascular disease occurs in low- and middle-income countries, including much of the Americas and the Caribbean, most scientific evidence originates from developed nations, such as the US and various regions of Europe (5,6). In 2011, Latin America accounted for only 4% of cardiovascular disease publications on MEDLINE, compared with 26% from the US/Canada and 42% from Europe, highlighting a significant disparity in knowledge generation (6,7). North America is the best-studied region, with an estimated six million HF patients and a projected prevalence of 3% by the year 2030 (4). In contrast, the estimated prevalence in South America is approximately 1%, with an annual mortality rate of 24.5% (8). Although some national studies and registries have attempted to characterize this population, the absence of a coordinated regional registry exacerbates the challenge of generating comprehensive data for the continent (9,10,11,12,13,14). Additionally, the data primarily come from Brazil and Argentina, the countries with the highest volume of epidemiological reports on cardiovascular disease in the American continent (15,16). This suggests underreporting in the rest of Latin America and the Caribbean, which is attributable to the lack of studies from other countries and to the deficiency in the quality of the available data (1,8,17).

Cardiovascular disease, including HF, remains one of the leading causes of mortality worldwide. The management established by clinical practice guidelines, known as optimal medical therapy (OMT), significantly improves survival in HF patients (18,19). OMT must include angiotensin-converting enzyme inhibitor (ACEI), angiotensin II receptor blocker (ARB), or angiotensin receptor-neprilysin inhibitor (ARNI); beta-blocker (BB); mineralocorticoid receptor antagonist (MRA); and sodium-glucose cotransporter 2 inhibitor (SGLT2i), and this quadruple intervention can reduce the relative risk of cardiovascular mortality by up to 73%. However, adherence to OMT is suboptimal, with reports showing adherence rates as low as 1% at the recommended doses in the current guidelines (18,19,20,21).

Given the above, the development of a continental HF registry (REGISTRO **AMER**ICANO DE **I**NSUFICIENCIA **C**ARDIA**C**A **A**MBULATORIA O **A**GUDAMENTE DE**S**COMPEN**S**ADA – AMERICCAASS) represents a pioneering initiative aimed at addressing knowledge gaps in the characterization and management of HF patients in the American continent, and at providing valuable data for the development of intervention strategies tailored to local realities (22). This study aims to characterize the HF population in the Americas and to evaluate the status of OMT in this population.

## Methods and Materials

The AMERICCAASS Registry is a prospective, observational, and multicenter study that was designed and coordinated by the board of directors of the Inter-American Council on Heart Failure and Pulmonary Hypertension (CIFACAH) of the Inter-American Society of Cardiology (SIAC). The Centro de Investigaciones Clínicas (CIC) of Fundación Valle del Lili (FVL) in Cali, Colombia, provided technical, logistical, and epidemiological support. Medical institutions from the Americas were invited to participate in the registry through their scientific entities or through a SIAC invitation. The Scientific Committee/Steering Committee is comprised of cardiologists affiliated to societies or associations that are members of the SIAC. For the registry, principal investigators may be internists, cardiologists, or HF subspecialists; sub-investigators may be nursing staff, general practitioners, internists, cardiologists, or HF subspecialists. Pre-established regional zones are coordinated by members of the Scientific Committee or other principal investigators, as determined by the Scientific Committee. Each national cardiovascular scientific society or association that is a member of SIAC was invited to designate a national coordinator for the registry to represent their respective country; this coordinator is a cardiologist delegated to CIFACAH. The AMERICCAASS Registry complies with the principles of the Declaration of Helsinki and received approval from the Comité de Ética en Investigación Biomédica (CEIB), the institutional ethics committee of FVL (13 November 2018/No. 313–2018). Additionally, the ethics committee of each participating institution approved the study protocol.

### Data Collection

The AMERICCAASS Registry included patients aged 18 and older (hospitalized or ambulatory) with a diagnosis of HF. Diagnosis of HF was based on information included in medical records that confirmed the diagnosis, and on receiving pharmacological treatment for this condition. The recruitment process began in April 2022 and concluded in November 2023, with a follow-up phase set to end in January 2025. A 12-month follow-up was planned for ambulatory patients, whereas 1-, 6-, and 12-month follow-ups were planned for hospitalized patients after discharge. Sociodemographic and clinical data were collected for each participant at the time of recruitment (ambulatory) or during hospital admission (hospitalized). Each participating medical institution was represented by a principal investigator and a sub-investigator who were responsible for entering data into the Research Electronic Data Capture (REDCap) electronic platform.

### Statistical analysis

For this subanalysis, the first 2,500 patients included in the registry between 2022 and 2023 were considered. Descriptive statistics were primarily used. The normality of quantitative variables was determined using the Shapiro-Wilk test. Quantitative variables were reported as means and standard deviations (SD) if normally distributed or as medians and interquartile ranges (IQR) otherwise. Categorical variables were presented as absolute frequencies and percentages.

OMT was defined in patients with left ventricular ejection fraction (LVEF) ≤49% as those receiving quadruple therapy, including ACEI/ARB/ARNI, BB, MRA, and SGLT2i. Pharmacological treatment data were reviewed focusing on three specific quadruple therapy combinations. Other potential combinations were not evaluated.

Combinations included:

1. ACEI + BB + MRA + SGLT2i
2. ARB + BB + MRA + SGLT2i
3. ARNI + BB + MRA + SGLT2i

These therapies were analyzed in relation to LVEF according to the current HF classification: HFrEF (HF with reduced ejection fraction, LVEF ≤ 40%), HFmrEF (HF with mildly reduced ejection fraction, LVEF 41–49%), and HFpEF (HF with preserved ejection fraction, LVEF ≥ 50%) (18). The Chi-square test was used to assess the association between different pharmacological groups and LVEF categories. All analyses were performed using R version 4.1.1 (R Foundation for Statistical Computing, Vienna, Austria) and RStudio version 1.4.1717. A map of the Americas illustrating the participating countries was designed using MapChart.

Detailed information on the methodology employed in the registry, data management, statistical procedures, and bioethical considerations has been described previously (23).

## Results

### Sociodemographic characteristics

**Figure 1:**
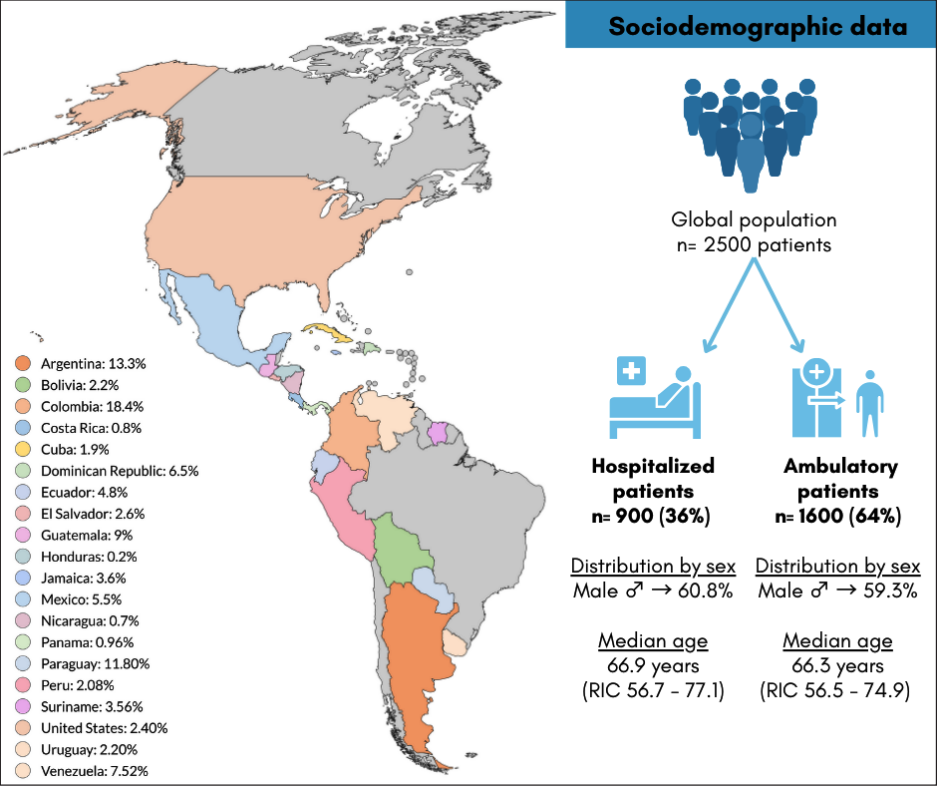
Distribution and sociodemographic characteristics of the patients.

The first 2,500 patients of the AMERICCAASS Registry were recruited from 82 medical institutions across 20 countries in the Americas (Argentina, Bolivia, Colombia, Costa Rica, Cuba, Dominican Republic, Ecuador, El Salvador, Guatemala, Honduras, Jamaica, Mexico, Nicaragua, Panama, Paraguay, Peru, Suriname, the US, Uruguay, and Venezuela). Of these, 900 (36%) patients were hospitalized and 1,600 (64%) patients were ambulatory. Males accounted for 60.8% of hospitalized and 59.3% of ambulatory patients. The median age was 66.9 years for hospitalized patients (IQR 56.7, 77.1) and 66.3 years for ambulatory patients (IQR 56.5, 74.9). The predominant race in both groups was Mestizo (60.8% vs. 61.3%), followed by White (22.7% and 24.6%, respectively) (Figure 1).

### Comorbidities and etiologies

Regarding comorbidities, most hospitalized patients had hypertension (HTN) (68.1%), dyslipidemia (38.1%), and diabetes mellitus (DM) (31.9%). Similarly, in ambulatory patients, hypertension (63.8%), dyslipidemia (45.3%) and DM (28.6%) were also highly prevalent. More than half of the patients had a body mass index (BMI) in the overweight or obese range (68.1% of hospitalized patients and 67.1% of ambulatory patients). Coronary artery disease (CAD) coexisted as a comorbidity in 31.6% of hospitalized and 37.5% of ambulatory patients (Table 1).

**Table 1:** Baseline characteristics of hospitalized and ambulatory patients.


VARIABLES	HOSPITALIZED	AMBULATORY

	N = 900 (36%)	N = 1,600 (64%)

COMORBIDITIES	n (%)	n (%)

Hypertension	613 (68.1)	1,020 (63.8)

Dyslipidemia	343 (38.1)	724 (45.3)

Diabetes mellitus	287 (31.9)	457 (28.6)

Coronary disease	284 (31.6)	600 (37.5)

Atrial fibrillation/atrial flutter	270 (30.0)	378 (23.6)

Overweight (BMI 25–29.9)	299 (38.2)	519 (39.3)

Obesity (BMI 30 to >40)	234 (29.9)	367 (27.8)

Previous COVID-19 infection	253 (28.1)	418 (26.1)

Chronic kidney disease	174 (19.3)	266 (16.6)

Hypothyroidism	93 (10.3)	172 (10.8)

Hyperthyroidism	8 (0.9)	11 (0.7)

Main etiology	n (%)	n (%)

Ischemic	314 (34.9)	602 (37.6)

Hypertensive	156 (17.3)	247 (15.4)

Valvular disease	108 (12.0)	137 (8.6)

Idiopathic	105 (11.7)	236 (14.8)

Chagas disease	57 (6.3)	61 (3.8)

Tachycardiomyopathy	47 (5.2)	127 (7.9)

Viral	15 (1.7)	23 (1.4)

Other	39 (4.3)	40 (2.5)

Lifestyle	n (%)	n (%)

**Smoking**	**345 (38.3)**	**604 (37.8)**

Previous	286 (82.9)	526 (87.1)

Current	59 (17.2)	78 (12.9)

**Alcohol**	**262 (29.0)**	**409 (25.5)**

1–2 times per week	130 (49.6)	163 (39.9)

3–5 times per week	27 (10.3)	41 (10.0)

5–7 times per week	19 (7.3)	12 (2.9)

Occasional-social	86 (32.8)	193 (47.2)

**Physical exercise**	**159 (17.6)**	**551 (34.4)**

1–3 days	129 (81.2)	398 (72.2)

4–7 days	30 (18.8)	153 (27.8)


COVID-19: Coronavirus Disease 2019.

The main HF etiologies among hospitalized patients were ischemic (34.9%), hypertensive (17.3%), and valvular (12%). In ambulatory patients, ischemic (37.6%) and hypertensive (15.4%) were also the most common etiologies, followed by idiopathic (14.8%). Other etiologies, such as Chagas disease, tachycardiomyopathy, or viral, accounted for smaller percentages in both groups (Table 1).

### Lifestyle

Most patients (71.6%) did not engage in physical exercise. Among those who exercised, 18.8% of hospitalized and 27.8% of ambulatory patients exercised 4–7 days per week. More than one-third of the patients had a history of smoking, with 38.3% of hospitalized and 37.8% of ambulatory patients, the majority being previous smokers in both groups (82.9% of hospitalized and 87.1% of ambulatory patients). Alcohol consumption was more common among hospitalized patients (29%) than ambulatory patients (25.5%). Among those who drank, hospitalized patients consumed alcohol more frequently, with 49.6% drinking one to two times per week, while 47.2% of ambulatory patients consumed alcohol occasionally or socially (Table 1).

### Clinical characteristics

Most patients had a reduced LVEF, with 60.7% of the hospitalized group and 58.5% of the ambulatory group. The predominant NYHA functional class differed between the two populations: Class III was the most common among hospitalized patients (46.6%), while Class II was prevalent in ambulatory patients (56.9%). Hospitalized patients experienced high rates of dyspnea on exertion (84.6%) and at rest (72.2%), while ambulatory patients had dyspnea on exertion (62.9%) and lower limb edema (28.1%) as their predominant symptoms (Table 2).

**Table 2:** Clinical characteristics of hospitalized and ambulatory patients.


VARIABLES	HOSPITALIZED	AMBULATORY

	N = 900 (36%)	N = 1,600 (64%)

NYHA	n (%)	n (%)

I	21 (2.4)	278 (18.7)

II	150 (17.0)	844 (56.9)

III	411 (46.6)	323 (21.8)

IV	300 (34.0)	39 (2.6)

No data	18	116

**LVEF**	**n (%)**	**n (%)**

≤40%	401 (60.7)	770 (58.5)

41–49%	133 (20.1)	321 (24.4)

≥50%	127 (19.2)	226 (17.2)

No data	239	283

**SYMPTOMS**	**n (%)**	**n (%)**

Dyspnea on exertion	761 (84.6)	1,006 (62.9)

Dyspnea at rest	650 (72.2)	173 (10.8)

Lower limp edema	622 (69.1)	450 (28.1)

Crackles	608 (67.6)	261 (16.3)


### Oral pharmacological treatment and OMT

At the time of hospital admission and recruitment, hospitalized patients were receiving the following medications (Table 3):

- BB (55.2%): mainly carvedilol (48.5%)
- MRA (38.9%): mainly spironolactone (93.4%)
- ARB (29.8%)/ACEI (18.8%)/ARNI (15.6%)
- SGLT2i (18.2%): mainly dapagliflozin (64%)
- Diuretics (51.6%): mainly furosemide (88.6%).

**Table 3:** Oral pharmacological therapy at the time of recruitment.


VARIABLES	HOSPITALIZED	AMBULATORY

	N = 900 (36%)	N = 1600 (64%)

PHARMACOLOGICAL THERAPY AT RECRUITMENT	n (%)	n (%)

**ARNI**	**140 (15.6)**	**428 (26.8)**

**ACEI**	**169 (18.8)**	**390 (24.4)**

Enalapril	143 (84.6)	310 (79.5)

Ramipril	14 (8.3)	34 (8.7)

Lisinopril	7 (4.1)	26 (6.7)

Captopril	3 (1.8)	13 (3.3)

Other	2 (1.2)	7 (1.8)

**ARB**	**268 (29.8)**	**428 (26.8)**

Losartan	158 (58.9)	218 (51.0)

Valsartan	55 (20.5)	86 (20.0)

Candesartan	13 (4.9)	54 (12.6)

Other	42 (15.7)	70 (16.4)

**BB**	**497 (55.2)**	**1,142 (71.4)**

Carvedilol	241 (48.5)	499 (43.7)

Bisoprolol	167 (33.6)	387 (33.9)

Metoprolol succinate	53 (10.7)	147 (12.9)

Nebivolol	22 (4.4)	92 (8.0)

Other	14 (2.8)	17 (1.5)

**MRA**	**350 (38.9)**	**902 (56.4)**

Spironolactone	327 (93.4)	805 (89.2)

Eplerenone	23 (6.6)	97 (10.8)

**SGLT2i**	**164 (18.2)**	**547 (34.2)**

Dapagliflozin	105 (64.0)	353 (64.5)

Empagliflozin	59 (36.0)	194 (35.5)

**Diuretic**	**464 (51.6)**	**858 (53.6)**

Furosemide	411 (88.6)	696 (81.2)

Thiazides	34 (7.3)	93 (10.8)

Others	19 (4.1)	69 (8.0)


At the time of recruitment, ambulatory patients were receiving the following medications (Table 3):

- BB (71.4%): mainly carvedilol (43.7%)
- MRA (56.4%): mainly spironolactone (89.2%)
- ARB (26.8%)/ACEI (24.4%)/ARNI (26.8%)
- SGLT2i (34.2%): mainly dapagliflozin (64.5%)
- Diuretics (53.6%): mainly furosemide (81.2%)

For the analysis of OMT, of 2,500 patients, 1,978 had LVEF information. Among them, 334 (16.9%) were receiving quadruple therapy, with most distributed between HFrEF (246 patients, 73.7%) and HFmrEF (56 patients, 16.8%), while a smaller proportion fell into HFpEF (32 patients, 9.6%) (Table 4).

- When analyzing quadruple therapy use within each HF subtype, 21% of all patients with HFrEF, 12.3% of all patients with HFmrEF, and 9% of all patients with HFpEF received it.
- The most frequent therapy included ARNI plus BB/MRA/SGLT2i in the overall analysis (72.5%). This was predominantly observed in patients with HFrEF (75.2%), and to a lesser extent in those with HFmrEF (69.6%) and HFpEF (56.3%). Despite those lower percentages, this combination remained the most prevalent therapy in the other two groups (HFmrEF and HFpEF).
- The therapy that included ARB plus BB/MRA/SGLT2i was the second most prevalent in the overall analysis (15.3%). It was mainly used in patients with HFpEF (40.6%) and HFmrEF (16.1%) and to a lesser extent in patients with HFrEF (11.8%).
- The combination that included ACEI plus BB/MRA/SGLT2i was the third most prevalent or least used OMT combination in the overall analysis (12.3%). It was more prevalent in HFmrEF (14.3%) and HFrEF (13%) than in HFpEF (3.1%).

**Table 4:** Distribution of quadruple therapy by LVEF.


	N = 1,978	≤40% N = 1,171	41–49% N = 454	≥50% N = 353	p < 0.001

	n (%)				

**Quadruple therapy**	**334 (16.9)**	**246 (21.0)**	**56 (12.3)**	**32 (9.0)**	

ARNI + BB + MRA + SGLT2i	242 (72.5)	185 (75.2)	39 (69.6)	18 (56.3)	

ARB + BB + MRA + SGLT2i	51 (15.3)	29 (11.8)	9 (16.1)	13 (40.6)	

ACEI + BB + MRA + SGLT2i	41 (12.3)	32 (13)	8 (14.3)	1 (3.1)	


## Discussion

HF is a disease with significant consequences for health, both regionally and globally. Given its high heterogeneity across regions, the implementation of initiatives like the AMERICCAASS Registry is crucial for identifying epidemiological and interventional differences between populations, which can help in developing targeted health policies (24). In this study, we included the first 2,500 patients recruited in the registry, describing sociodemographic, clinical, and therapeutic aspects of HF patients in the Americas and providing a realistic overview of the region.

### Sociodemographic characteristics

The predominance of male sex and Mestizo race, along with the reported mean ages, reflects the demographic behavior of HF in the nations studied. Globally, the age-standardized prevalence of HF is similar between sexes, although slightly higher in males (24,25). The incidence, on the other hand, is lower in females, especially in HFrEF (25). The findings of this study align with these trends, mirroring international data. These sex-based differences may be attributed to the distribution of cardiovascular risk factors between men and women (26,27).

Traditionally, HF has been associated with older age groups. A 2022 systematic review reported a higher prevalence in patients aged over 50 years (28). Similarly, the PULSE study reported a mean age of 75 years for HF patients and a higher incidence among those aged over 85 years (29). The findings of this study show median ages over 60 years for both hospitalized and ambulatory groups, which are consistent with scientific evidence and global trends. However, recent European studies show a rising incidence of HF in patients under 50 years, attributed to modifiable risk factors, such as obesity and smoking (30,31,32,33). This trend underscores the need for targeted prevention and management strategies for younger patients.

### Comorbidities and etiologies

Our findings show a significant prevalence of comorbidities, including HTN, dyslipidemia, CAD, and DM, in both hospitalized and ambulatory HF patients. Ischemic and hypertensive etiologies were identified as the main causes of HF in both groups. This is consistent with current scientific evidence that highlights these conditions among the leading predisposing factors for HF (25). The INTER-CHF study reported high rates of HTN (73.6%), dyslipidemia (48.7%), DM (21.9%), and myocardial infarction (18.3%) among South American HF patients. The same study found that ischemic (25%) and hypertensive (21%) were the predominant etiologies of HF in this region (16,25). Similarly, the REPORT-HF study found HTN (68%) to be the most common comorbidity, with CAD (33%) and type 2 diabetes (31%) also being prevalent in Central and South America. This study also found that ischemic and hypertensive etiologies were the leading causes of HF in the Americas (34). Both studies reported high frequencies of these etiologies and comorbidities on a global scale (16,34). The high prevalence of these conditions underscores the need for a comprehensive strategy to optimize HF management by addressing key comorbidities.

Our study also identified a high proportion of overweight and obese patients, consistent with the existing evidence. The REPORT-HF study reported a median BMI in the overweight range and a 10% prevalence of obesity in patients from Central and South America, which was lower than 49% prevalence reported from North American populations (34). Similarly, the INTER-CHF study found a mean BMI in the overweight range for the HF patients from South America, with comparable results in other regions (16). These findings emphasize the need for comprehensive patient management, including effective weight control strategies.

### Lifestyle

Our findings reveal a higher prevalence of unhealthy habits among HF patients, characterized by low levels of physical activity and high rates of smoking and alcohol consumption, consistent with scientific literature. Sedlar et al. observed low adherence to exercise (39%), primarily related to a lack of self-care knowledge (35). Smoking and alcohol consumption, recognized risk factors for cardiovascular diseases, are strongly associated with higher HF incidences and hospitalization rates in these patients (36,37,38). For instance, a study involving 989,080 hospitalized HF patients found that 15% had substance use disorders: 12.1% related to tobacco and 3.5% to alcohol. The highest prevalence was observed in those aged 45 to 55, potentially correlating with the rising incidence of HF in younger populations (39). The INTER-CHF study also demonstrated higher rates of tobacco and alcohol consumption in Latin America compared with other territories (16).

The observations from the AMERICCAASS cohort and related studies suggest suboptimal adherence to non-pharmacological recommendations for HF management, highlighting key intervention areas across North, Central and South America. Targeted education and structured programs, such as smoking cessation, alcohol reduction, and supervised exercise, could enhance adherence and improve quality of life and health outcomes, as habits like regular exercise have been shown to reduce hospitalization and mortality in HF patients (40).

### Clinical characteristics

The results of this study show a predominance of NYHA classes II and III in ambulatory and hospitalized patients, respectively. Additionally, most patients had HFrEF, regardless of their status. These findings reflect the severity of HF in the Americas and align with existing literature. The INTER-CHF study reported similar trends in South America, Africa, Asia, and the Middle East (16). The REPORT-HF study also found a predominance of NYHA class II and III patients in all the regions evaluated, including Central and South America. The same study revealed a high prevalence of patients with HFrEF globally, accounting for 50% of the studied population (34). However, this contrasts with studies such as I PREFER, which reported a higher prevalence of patients with LVEF (>45%) in Latin America, associated with a predominance of NYHA classes other than III/IV (70%) (41).

### Oral pharmacological treatment and OMT

The percentage use of BBs, MRAs, ARBs/ACEI/ARNI, and SGLT2i in the AMERICCAASS Registry demonstrates alignment with current scientific evidence, which supports the use of these pharmacological groups in combination based on LVEF (19). However, the use of SGLT2i and MRAs is lower compared with the other pharmacological groups, highlighting the need for targeted strategies to increase their uptake in eligible patients.

The evaluation of pharmacological combinations also reveals a trend in management that aligns with current clinical practice recommendations, as quadruple therapy was primarily observed in patients with HFrEF (73.7%) and HFmrEF (16.8%). Its prescription for patients with HFrEF is widely recommended in the leading HF guidelines, which advocate for a foundational approach in treating this subpopulation, with a preference for ARNI over ARBs and ACEI, as observed in previous results (18,19,42,43). In patients with HFmrEF, a significant use of quadruple therapy was observed, with a preference for management with ARNI. Although the scientific evidence is less robust, it suggests a potential benefit of this therapy in this subgroup of patients, particularly in terms of mortality and hospitalizations (43).

Quadruple therapy was less commonly used among patients with HFpEF, reflecting the individualized treatment approaches recommended by clinical guidelines. However, 9.6% of the overall use of quadruple therapy was observed in this group, and 9% of all HFpEF patients received such treatment, particularly ARB-based combinations, possibly deviating from clinical recommendations. In this subgroup, the prescription of SGLT2i is prioritized, as it is the pharmacological group with the highest level of evidence (43). Nonetheless, recent studies suggest a potential benefit of MRAs and ARNI in this population, which may justify their prescription. These recommendations, however, only apply to patients with LVEF lower than 60% (44). Moreover, the recent FINESART-HF trial demonstrated that finerenone significantly reduces the rate of composite outcomes, including worsening HF events and cardiovascular mortality, in patients with HFmrEF and HFpEF. While the data used in this study precedes these findings, this recent evidence could bolster their relevance and could highlight the potential application of MRAs in clinical practice (45,46). By contrast, the evidence regarding the use of BBs in this group is more limited and does not indicate clear benefits (43). This discrepancy may suggest deviations from current clinical guidelines, although further research is necessary to draw definitive conclusions.

While the findings of this study on prescribing patterns partially align with current recommendations, adherence to OMT remains low, with only 21% of patients with HFrEF receiving guideline-recommended management. These results, however, are higher than some existing reports that evaluate adherence to OMT, which show very low usage rates. Anderson et al., for example, found that approximately 1% of patients in this subgroup receive the recommended pharmacological management (47). In contrast, the CHAMP-HF Registry found a 22.1% usage rate of ARBs/ACEI/ARNI, BBs, and MRAs, although only 1% of patients were receiving this management at optimal doses (47,48). Another study found that 18% of their HF patients in cardiac rehabilitation were receiving OMT (49). Similarly, the findings of this study for HFmrEF showed a low usage of OMT, with only 12.3% receiving quadruple therapy.

These findings demonstrate that initiating and adhering to OMT are a healthcare challenge, especially in low- and middle-income countries (50). The minimal increases in utilization rates between the years 2013 and 2019 reflect this issue (51). Multiple barriers contribute to these difficulties, including clinical factors such as early discharge pressure, misunderstanding of therapy by patients, and knowledge gaps on evidence-based recommendations by physicians (50,52). Moreover, advances in medical knowledge may outpace the ability of healthcare systems to implement them. Additionally, the underrepresentation of certain populations in clinical trials may potentially impact adherence, as inclusion criteria may differ from real-world practice. However, this is unlikely to fully explain the substantial gaps in OMT application, as inclusion criteria are typically fundamental eligibility requirements (53).

Economic factors, especially limited access to healthcare and lack of health insurance, also play a role in these dynamics (50,52). Patient costs, particularly prescription copayments, may impact the implementation of OMT (53). Additionally, social disparities regarding sex or ethnicity may affect the prescription of certain pharmacological groups, further hindering OMT implementation. For instance, the CHAMP-HF Registry found that women and Hispanic patients were less likely to receive ACEI, ARBs, and ARNI than other groups (48,50,54).

Although implementing OMT presents several challenges, various strategies, such as educational sessions, reminder systems, self-care support initiatives, and telemonitoring, have been studied for their effectiveness in improving OMT adherence in HF. A meta-analysis of 55 randomized controlled trials found that adherence-enhancing strategies increased adherence by 10%. Interestingly, 31 trials demonstrated their effectiveness in promoting lifestyle modifications, reinforcing the need to address poor adherence to non-pharmacological recommendations, as highlighted in previous findings (55). Notably, these interventions have also been shown to reduce mortality and hospitalizations, strengthening their clinical importance (55,56). Finally, digital interventions that collect data beyond structured care episodes could help to optimize pharmacological therapy in these patients; however, further research is needed (57).

## Strengths and limitations

This study presents findings that enhance the current scientific evidence. The analysis of 2,500 patients with HF across the Americas increases the reliability and validity of the results obtained. Additionally, it provides comprehensive and representative views of HF in various regions of North, Central, and South America and the Caribbean, providing a foundation for future research. The multicentric and prospective approach allows for the collection of uniform and up-to-date data, reducing retrospective bias and improving the quality of information regarding the clinical characteristics and management of HF. Additionally, the use of REDCap for data collection and storage ensures efficient, secure, and confidential information management.

This design has certain limitations. Regions with fewer resources and limited access to healthcare may be underrepresented, potentially leading to an overestimation of data from other regions and affecting generalizability. OMT was considered for any patient prescribed the four pharmacological groups recommended by clinical guidelines, without accounting for contraindications or dose adherence, limiting the depth of the analysis possible. Future studies should address this by incorporating a more detailed evaluation of dosage adherence. Additionally, some variables rely on patient self-reporting, introducing the risk of information bias. Countries that had not yet participated in the registry at the time of analysis, including Puerto Rico, Canada, Chile, and Brazil, were excluded. Notably, the absence of Brazil, a major contributor to Latin American healthcare research with a vast and diverse healthcare system, may limit the generalizability of the findings of this study. Furthermore, the lack of participation from certain countries with extensive rural and underserved areas may further limit the representativeness of the results. Low participation was also noted in both resource-limited regions and larger countries, as registry involvement is voluntary.

## Conclusions and Future Perspectives

Our findings reveal that HF sociodemographic and clinical characteristics in America are similar to other international studies, highlighting the marked prevalence of patients with HFrEF. Additionally, the high prevalence of unhealthy lifestyle habits is noteworthy. The use of OMT is relatively low in the evaluated population, which may reflect suboptimal patient adherence or gaps in medical prescribing practices, indicating a deviation from the recommendations of clinical guidelines regarding quadruple therapy. It is essential to delve into the underlying causes of these deviations to develop local, national, and regional interventions that can improve adherence to OMT and, consequently, can optimize health outcomes.

## Data Accessibility Statement

The data presented in this study are available in the article.
